# Malaria in the Post-Partum Period; a Prospective Cohort Study

**DOI:** 10.1371/journal.pone.0057890

**Published:** 2013-03-13

**Authors:** Machteld E. Boel, Marcus J. Rijken, Tjalling Leenstra, Aung Pyae Phyo, Mupawjay Pimanpanarak, Naw Lily Keereecharoen, Stephane Proux, Natthapon Laochan, Mallika Imwong, Pratap Singhasivanon, Nicholas J. White, Rose McGready, François H. Nosten

**Affiliations:** 1 Shoklo Malaria Research Unit, Mae Sot, Tak, Thailand; 2 Center for Tropical Medicine and Travel Medicine, Academic Medical Centre, University of Amsterdam, Amsterdam, The Netherlands; 3 Faculty of Tropical Medicine, Mahidol University, Bangkok, Thailand; 4 Centre for Tropical Medicine, Nuffield Department of Clinical Medicine, University of Oxford, Oxford, United Kingdom; Université Catholique de Louvain, Belgium

## Abstract

**Background:**

Several studies have shown a prolonged or increased susceptibility to malaria in the post-partum period. A matched cohort study was conducted to evaluate prospectively the susceptibility to malaria of post-partum women in an area where *P.falciparum* and *P.vivax* are prevalent.

**Methods:**

In an area of low seasonal malaria transmission on the Thai-Myanmar border pregnant women attending antenatal clinics were matched to a non-pregnant, non-post-partum control and followed up prospectively until 12 weeks after delivery.

**Results:**

Post-partum women (n = 744) experienced significantly less *P.falciparum* episodes than controls (hazard ratio (HR) 0.39 (95%CI 0.21–0.72) p = 0.003) but significantly more *P.vivax* (HR 1.34 (1.05–1.72) p = 0.018). The reduced risk of falciparum malaria was accounted for by reduced exposure, whereas a history of *P.vivax* infection during pregnancy was a strong risk factor for *P.vivax* in post-partum women (HR 13.98 (9.13–21.41), p<0.001). After controlling for effect modification by history of *P.vivax*, post-partum women were not more susceptible to *P.vivax* than controls (HR: 0.33 (0.21–0.51), p<0.001). Genotyping of pre-and post-partum infections (n⊕ = ⊕10) showed that each post-partum *P.falciparum* was a newly acquired infection.

**Conclusions:**

In this area of low seasonal malaria transmission post-partum women were less likely to develop falciparum malaria but more likely to develop vivax malaria than controls. This was explained by reduced risk of exposure and increased risk of relapse, respectively. There was no evidence for altered susceptibility to malaria in the post-partum period. The treatment of vivax malaria during and immediately after pregnancy needs to be improved.

## Introduction

For nearly a century it has been known that pregnant women are more susceptible to malaria than non-pregnant women of a similar age[1]. Less is known about susceptibility in the post-partum period. Some studies report prolonged and increased susceptibility to malaria comparable to that in pregnancy[2], [3], while others report spontaneous clearance of *P.falciparum* parasites within 24 hours of delivery[4], [5]. A recent review including 11 studies suggests a transition phase after delivery in which the susceptibility gradually returns to pre-pregnancy levels[6]. *P.falciparum* genotyping suggested persistence of parasites acquired during pregnancy into the post-partum period[3], [7]. Submicroscopic infections and ineffective malaria treatment during pregnancy increase the risk of recrudescence in the post-partum period[6], [7]. In contrast there are no reports on *P.vivax* in the post-partum period.

We conducted a matched cohort study to determine prospectively whether post-partum women are at increased risk of *P.falciparum* and *P.vivax* malaria in the 12 weeks following delivery.

## Materials and Methods

### Ethics statement

Ethics approval was obtained from the ethics committee at the Faculty of Tropical Medicine, Mahidol University Bangkok, Thailand (MUTM 2007-023) and at Oxford Tropical Medicine Ethical Committee, Oxford University, England (Code 002-07) and yearly renewed. All participants gave written, informed, or thumb print if illiterate, consent before enrolment in the study.

### Study population and Antenatal care

The Shoklo Malaria Research Unit (SMRU) antenatal clinics (ANC) are located on the north western border of Thailand where there is low seasonal transmission of both multidrug resistant *P.falciparum* and increasingly resistant *P.vivax* malaria[8]. There is currently no safe and effective drug for chemoprophylaxis or intermittent preventive treatment (IPT) in pregnancy. Early detection and treatment of malaria with an artemisinine-based combination therapy (ACT) has been deployed in the area since 1994[9], [10]. For pregnant women active weekly detection and early treatment is the only method to prevent maternal death from malaria[8]. Anemia prophylaxis or treatment is provided to all women. All confirmed episodes of malaria are treated according to international guidelines[11], [12]: *P.falciparum* is treated with mefloquine + artesunate in non-pregnant individuals, and with quinine (first trimester) or artesunate + clindamycin in pregnant individuals. *P.vivax* is treated with chloroquine. Primaquine is not used in the study population, because it is contraindicated during pregnancy and lactation. Also in the non-study population primaquine is not widely used, because of high rates of G6PD deficiency. The prevalence of HIV and syphilis is less than 0.5% in this population[13]. The majority of women presented in this study are migrant workers from Myanmar, who don’t have easy access to the Thai health system. Therefore it is unlikely that women with fever would present at other clinics than at SMRU clinics, where medical care is free of charge.

### Procedures

All women attending the ANC from November 2007 to September 2009 were asked to participate. At inclusion a socio-economic questionnaire was conducted and malaria history documented. Before delivery each woman was asked to help find a female friend or relative of similar age (+/−10 years), living within 200 meters, who was not pregnant or recently delivered who would be a control for the 12 weeks post-partum period. Pregnancy in the control women was excluded by urine β-HCG test (Bioline HCG strip) at enrolment and again at week 12. Weekly smears, blood spots on Whatman 3 M filter paper and symptom questionnaires on residential location, insecticide treated bed nets (ITN) usage and self treatment with antimalarial drugs and monthly hematocrit were obtained from both groups for 12 weeks.

Blood smears (thin and thick films) were stained with Giemsa and read directly on site for 200 fields before being declared negative. Quality control by experienced technicians revealed a negative predictive value of the routine lab technicians of 97.1% (232/239), and a positive predictive value 97.7% (338/346). Blood spots on filter paper were dried and stored for later analysis.

### Genotyping

Pregnancy and delivery blood samples of women who were malaria negative by microscopy during pregnancy, but had malaria in the post-partum period were screened for submicroscopic infection. For parasite detection the genes encoding the small subunit ribosomal RNA (ssRNA) were amplified, then species-specific primer-pairs were used in the nested round[14]. For *P. falciparum* pre-and post-partum 3-locus genotypes (MSP-1, MSP-2 and GLURP) were compared[15], [16]. Sub-microscopic infections were not counted as malaria infections in the incidence and risk calculations, and not treated, because analysis was performed later.

### Sample size calculation

A sample size of 713 post-partum women and 713 controls was required to allow the detection of an increase in the cumulative proportion of women with malaria during the 12 weeks from 5% in the control women to 10% in the women post-partum with 90% power, 95% confidence, and 15% drop out.

### Statistical analysis

Data were analysed in SPSS for Windows version 18.0, STATA version 11 and R version 2.12. Baseline comparisons were made using Student's t-test and Mann–Whitney U-test. Proportions were compared using the Chi-square test. For incidence-rates and time-to-event analysis the time to the first microscopically confirmed *P.vivax* or *P.falciparum* infection in the 12 week period was calculated, after which the woman was censored. If a *P.falciparum* infection preceded a *P.vivax* infection, three weeks were subtracted from the weeks for *P.vivax* incidence-rate, because the antimalarial treatment of *P.falciparum* prevents *P.vivax.* If a *P.vivax* infection preceded a *P.falciparum* infection, the *P.falciparum* incidence-rate was unchanged, because chloroquine is ineffective against *P.falciparum* in this area[17]. Mixed infections (*P.vivax* and *P.falciparum* infection at the same time) were counted in both incidence-rates. The incidence of malaria in post-partum women was compared to the incidence in controls using Cox-proportional hazards regression with shared frailty[18] to adjust for the non-independence of observations resulting from pair-matching. Though matching was expected to preclude confounding, residual confounding by factors not used for matching was evaluated by assessing the change in the main effect following inclusion of these factors in the Cox-regression model. The time to the first symptomatic non-malaria febrile episode was compared between post-partum and control women using above Cox-regression methods separately for the first and the last 6 weeks. A linear mixed regression model was used to model mean change in hematocrit during follow-up. A model including random intercepts and slopes for individuals best fitted the data. The functional form of mean change in hematocrit over time was explored and was best fit as a linear spline with a single knot at 4 weeks.

If a woman was absent from planned follow-up she was considered to be malaria and fever negative, assuming that most people become symptomatic when infected with malaria in this area,[19] and present to medical attention. Women were considered as lost to follow-up if follow-up was discontinued and the woman did not return within the study period. From women who became pregnant during the post-partum study, the date of conception was back-calculated by use of ultrasound and only data collected before the date of conception was used for analysis.

## Results

**Figure 1:**
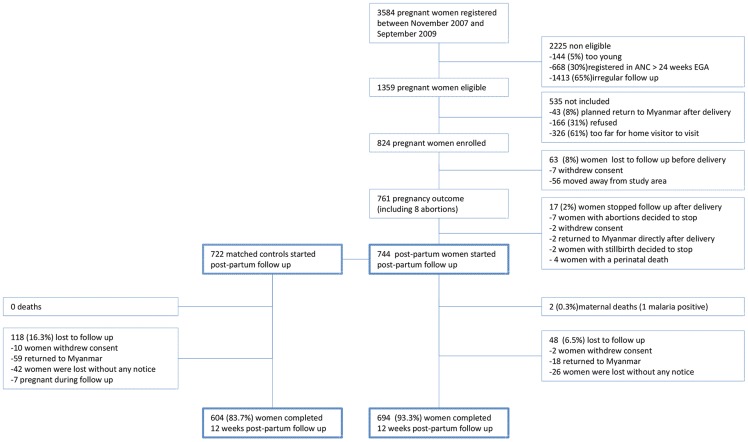
Schematic flow diagram of the enrolment process and study follow-up during pregnancy and post-partum.

From November 2007 until September 2009, 824 women were enrolled during pregnancy, of whom 744 started the post-partum follow-up. Of these 744 women, 722 (97%) had a matched control (Fig. 1). The groups were comparable except that as expected post-partum women had a slightly higher median number of previous pregnancies, and more often reported a history of malaria in the previous 9 months (Table 1). Over 90% of women reported sleeping under an ITN.

**Table 1:** Characteristics of post-partum women and controls.

	Post-partum (n = 744)	Controls (n = 722)	P-value
Age (years)	27 (23–33), [18–48]	27 (22–33), [18–51]	NS
<21 years	145 (19.5)	130 (18.0)	NS
Parity (P)	3 (1–4), [1]–[14]	2 (1–3), [0–13]	<0.001
Nullipara	0	172 (23.8)	<0.001
Primipara (P = 1)	196 (26.4)	131 (18.1)	<0.001
Multipara (P>1)	548 (73.6)	419 (58.0)	<0.001
Years of schooling	3 ± 3	3 ± 3	NS
Literate	448 (60.3)	472 (65.4)	0.046
Smoke	177 (23.7)	172 (23.7)	NS
Use of bed nets	678 (91.1)	673 (93.2)	NS
Distance to the clinic, minutes	38 ± 29	38 ± 27	NS
*Socio-economic status:*			
Lower group	193 (25.9)	213 (29.6)	NS
Middle group	366 (49.2)	350 (48.5)	NS
Upper group	185 (24.9)	159 (22.0)	NS
History of *P.falciparum*	117 (15.7)	22 (3.0)	<0.001
History of *P.vivax*	244 (32.8)	25 (3.5)	<0.001

Data presented as mean ± standard deviation, median (inter quartile range), [min-max] or n (%). NS; not significant

Women in the control group were more likely to work away from home, in the field or forest (Table 2). A higher proportion of post-partum women (>90%) completed follow-up compared to controls (>80%). Few women reported self-treatment of fever with antimalarials; 6/1466 (0.4%): in two malaria parasites were detected, the other 4 women remained malaria negative. Two post-partum women died during the study period. One died at home 11 days after delivery from dysentery. The other woman died 24 days after delivery from severe malaria in a hospital in Myanmar.

**Table 2:** Observations during study follow-up in post-partum mothers and controls.

	Post-partum (n = 744)	Controls (n = 722)	P-value
Follow-up completed	694 (93.3)	604 (83.7)	<0.001
No absence during 12 weeks	349 (46.9)	180 (24.9)	<0.001
Absent 1 or 2 times	280 (37.6)	274 (38.0)	NS
Absent >6 times	26 (3.5)	53 (7.3)	<0.001
Slept without a bed net at least 1 night during follow-up	66 (8.9)	49 (6.9)	NS
Slept at least 1 day in the field/forest during follow-up	83 (11.2)	96 (13.3)	NS
Worked at least 1 day in the field/forest during follow-up	83 (11.2)	282 (39.1)	<0.001
Became pregnant during follow-up	4 (0.5)	28 (3.9)	<0.001
Reported self treatment with antimalarials	2 (0.3)	4 (0.6)	NS

Data presented as n (%). NS; not significant

### Malaria

The proportion of post-partum women with any malaria in the 12 week follow up period was 168/744 (22.6%) compared to 138/711 (19.4%) of the controls, p = 0.140. The incidence of *P.falciparum* malaria was lower in the post-partum women compared to control women, but the reverse was true for *P.vivax* episodes (Table 3). The crude hazard ratio (HR) in post-partum women compared to controls for *P.falciparum* and *P.vivax* was 0.39 (0.21–0.72) p = 0.003 and 1.34 (1.05–1.72) p = 0.018, respectively (Table 4). Occupational exposure and a history of *P.falciparum* in the previous 9 months were both associated with an increased risk of *P.falciparum* malaria (p<0.001 for both, Table 4). After adjusting for exposure (use of an ITN, sleeping or working outside in the field/forest) the hazard ratio for *P.falciparum* was attenuated and no longer significant (p = 0.260, Table 5).

**Table 3:** Incidence-rates of *P.vivax* and *P.falciparum* during the 12 weeks post-partum follow-up.

		No. of woman-weeks of follow up	No. of first episodes within 12 weeks	Incidence-rate of first episodes per 1000 woman–weeks (95% CI)	P-value
*P.falciparum*	Post-partum mothers	8538	23	2.7 (1.75–3.98)	0.022
	Controls	7609	36	4.7 (3.36–6.48)	
*P.vivax*	Post-partum mothers	7662	155	20.2 (17.2–23.6)	0.016
	Controls	7160	110	15.4 (12.7–18.4)	

**Table 4:** Hazard ratios for different factors for *P.falciparum* and *P.vivax* during the 12 weeks post-partum follow-up.

	P.falciparumHazard ratio (95% CI)	P-value	P.vivaxHazard ratio (95% CI)	P-value
Being a post-partum woman	0.39 (0.21–0.72)	0.003	1.34 (1.05–1.72)	0.018
Multipara	0.33 (0.16–0.71)	0.005	1.11 (0.85–1.45)	0.44
<21 years	1.28 (0.67–2.43)	0.46	1.38 (1.02–1.39)	0.036
Smoke	1.06 (0.57–1.96)	0.86	1.35 (1.02–1.80)	0.038
Illiterate	0.61 (0.27–1.42)	0.26	1.23 (0.95–1.60)	0.11
Not used the bednet some nights	0.94 (0.33–2.70)	0.91	0.84 (0.51–1.42)	0.53
Slept outside some nights	1.83 (0.91–3.69)	0.09	1.30 (0.89–1.89)	0.17
Worked in the field/forest	2.41 (1.40–4.15)	0.002	1.10 (0.82–1.46)	0.52
History of P.falciparum	3.86 (2.12–7.05)	<0.001	3.12 (2.27–4.27)	<0.001
History of P.vivax	2.12 (1.21–3.71)	0.009	6.58 (5.14–8.43)	<0.001
History of P.vivax recurrences	0.67 (0.27–1.63)	0.374	2.53 (1.66–3.87)	<0.001
History of any malaria	2.17 (1.26–3.73)	0.005	5.64 (4.39–7.24)	<0.001

**Table 5:** Adjusted hazard ratios for the effect of being a post-partum woman on *P.falciparum* and *P.vivax*.

	Hazard ratio (95% CI) for *P.falciparum*	P-value	Hazard ratio (95% CI) for *P.vivax*	P-value
Crude risk being a post-partum woman	0.39 (0.21–0.72)	0.003	1.34 (1.05–1.72)	0.018
Adjusted for age, parity, illiteracy, smoke	0.58 (0.34–1.00)	0.050	1.36 (1.06–1.75)	0.017
Adjusted for exposure (occupation, sleeping outside, bed net use)	0.72 (0.40–1.27)	0.260	1.47 (1.13–1.92)	0.004
Adjusted for history of *P.falciparum*	0.36 (0.20–0.65)	<0.001	na	na
Adjusted for history of *P.vivax*	na	na	0.47 (0.34–0.64)	<0.001

na; not applicable.

History of *P.vivax* malaria was a strong risk factor for *P.vivax*: HR 13.98 (9.13–21.41) in post-partum and 5.04 (2.70–89.38) in control women (p<0.001 for both). In addition, history of *P.vivax* was an effect modifier of the observed effect of being post-partum on *P.vivax* risk (interaction-term p = 0.008), requiring the estimation of stratum specific effects of being post-partum on *P.vivax* risk[20]. In women with a history of *P.vivax*, the risk for *P.vivax* in the study period was not different between post-partum women and control women, HR 0.92 (0.50–1.69), p = 0.783. In the 500 women who did not have a history of *P.vivax*, the risk of *P.vivax* in post-partum women was actually lower than that in the control group (HR: 0.33 (0.21–0.51), p<0.001. There was no difference in the time to the first *P.vivax* in women developing *P.vivax* during the 12 week study-period: 39 days (± 22, range 2–86 days) for post-partum women and 38 days (± 22, range 4–84 days) for the controls, p = 0.594.

Of the 13 paired pregnancy and post-partum *P.falciparum* infections: DNA for genotyping could not be amplified in 2, and one pair was incomplete. All 10 genotyped infections in the post-partum period were with different genotypes to those in pregnancy, suggesting these were newly acquired infections. Among 26 post-partum women who experienced no infections during pregnancy but had their first infection in the post-partum period, one (3.8%) had a submicroscopic infection in her peripheral blood at delivery.

Post-partum women with *P.falciparum* were more likely to have a documented fever than controls (RR 3.24 (1.11–9.43), p = 0.026), and in those with fever (n = 35) the mean temperature (Standard Deviation (SD)) was slightly higher in post-partum mothers than in the controls (38.4°C (0.7) vs 38.2°C (0.8), p = 0.46). Also the geometric mean parasitemias (/ µl) for *P.falciparum* and *P.vivax* were slightly higher in post-partum women, but not significantly (p = 0.85 and 0.57 respectively).

### Fever (non-malaria)

Post-partum women experienced more febrile episodes (excluding malaria and clearly post-partum related morbidities e.g. mastitis, endometritis and puerperal sepsis) in the first 6 weeks (HR 2.94 (1.70–5.10), p<0.001) after delivery but not more than controls in the second 6 weeks (HR 0.93 (0.54–1.56), p = 0.782) of the follow-up. In febrile cases the mean temperature (SD) was 38.1°C (0.6) in the post-partum women compared to 37.9°C (0.5) in the controls, p = 0.039. In 87/144 (60.4%) of cases no clinical diagnosis of the febrile illness could be made and all resolved with symptomatic treatment.

### Anaemia

Directly after delivery the mean (SD) hematocrit in post-partum women was 35.1% (5.6), 2.88% lower (1.91–3.84) than in controls (37.9% (3.9)), p<0.001. Although the mean hematocrit in post-partum women increased during follow-up, it remained slightly lower at the end of follow-up; 0.66% hematocrit (0.01–1.31), p = 0.048. Post-partum women with *P.vivax* malaria during follow-up had a 0.62% (−0.14–1.27) lower rate of hematocrit increase per week following malaria, compared to post-partum women without *P.vivax*.

## Discussion

In this cohort study conducted in an area of low seasonal malaria transmission, post-partum women were not at greater risk of malaria compared to matched controls. The main risk factor for *P.falciparum* malaria was the occupational exposure i.e working in the fields or in the forest where exposure to the main vectors (*Anopheles* (*An.*) *dirus*, and *An. minimus*) is higher. As expected women who have just delivered a baby are less involved in field work away from their domestic residence and therefore less exposed to infective bites. For *P.vivax* the main risk of a parasitaemic episode post-partum was a recent history of vivax malaria. This strongly suggests relapse of hypnozoites from the liver, and this is substantiated by two studies from the same area [21], [22]. Recrudescent infections due to chloroquine resistance are unlikely to be responsible for the observed risk, because the 28-day cure rate of chloroquine in the non-pregnant population is still above 90%[23]. After stratification for history of vivax malaria, the post-partum period was not associated with a higher susceptibility to *P.vivax* malaria. Indeed after adjusting for the malaria history and the other confounders, the post-partum period was associated overall with a relative protection against malaria, probably because of the lower exposure to infected vectors.

It has been proposed in the past that intensive physical strain can cause a relapse of *P.vivax* [24]. But whether delivery can provoke a relapse of *P.vivax* seems unlikely: the risk of *P.vivax* infection in the 12 week period among women with *P.vivax* in the history, and the mean time to the first *P.vivax* infection after delivery was the same as in the control group.

*P.falciparum* genotyping in this cohort suggests that *P.falciparum* during pregnancy is cured by current treatment (quinine in combination with clindamycin in the first trimester, artesunate with clindamycin in the 2^nd^ and 3^rd^ trimester), as 10/10 pregnancy-post-partum paired samples showed new infection after delivery. This is in contrast with reports from Mozambique and Gabon, where 30–50% of infections after delivery were with the same genotype as infections at delivery[3], [7]. This may reflect poor treatment efficacy; at the time the study was conducted in Mozambique, *P.falciparum* resistance to the antimalaria used (sulfadoxine-pyrimethamine) was high and in Gabon quinine was given which is much less effective than artemisinin combination therapy. The higher malaria transmission in Africa results in greater carriage of submicroscopic infections into the post-partum period; in Mozambique 66/320 (20.6%) of women carried submicroscopic peripheral infection at delivery[7], compared to 1/26 (3.8%) in our study.

Contrary to the increased risk for *P.falciparum* malaria after delivery reported from Senegal[2], the data presented here does not suggest any alteration in clinical features. There are several possible explanations for the divergent conclusions: firstly the study in Senegal took place in an area of resistance to deployed drugs and high levels of sub-patent *P.falciparum* were reported[25], [26], [27], and women with asymptomatic low parasitemia were not treated during pregnancy making it likely that the women carried the parasites into the post-partum period. Secondly there is the issue of misdiagnosis. We found post-partum women to have more febrile episodes (irrespective of malaria) in the first month after delivery than controls. When clinical malaria is an endpoint (as in the study in Senegal) in an area of intense transmission, women after delivery may be more likely to have fevers and coincidental parasitemia. This is supported by the finding in the study in Senegal that there was no increased incidence in asymptomatic *P.falciparum* infections after delivery.

In post-partum women as well as in controls the usage of ITNs was high. The few women who reported that they had not been sleeping under their ITN did not have an increased risk of getting malaria. Although the numbers were small, this is consistent with previously reported data in this area that did not show a significantly protective effect on parasitaemia of ITNs in pregnancy, possibly due to the main vectors for malaria in this area which prefer to bite at sunset and sunrise, when adults are not sleeping under their bed net[28].

There are several limitations of this study: The history of malaria in the previous 9 months in the post-partum women was obtained from the pregnancy records with weekly screening results reported, whilst the history of control women was obtained more passively, from the patients handheld medical record if available, or from the patients recall possibly resulting in under-reporting. Another limitation was that the heterogeneity of transmission in the study area was not assessed, but the matching of control women on residency within 200 meters of the pregnant woman should have controlled for this.

In conclusion, in this area where *P.falciparum* infection during pregnancy is detected and treated early and aggressively and submicroscopic infection is rare, post-partum women are less susceptible for *P.falciparum* than controls, but this is explained by lower exposure. For *P.vivax* the treatment during pregnancy is limited to the blood stage infection because primaquine is contra-indicated in pregnancy. Relapse rates are high and commonly present in the post-partum period. Treatment for the *P.vivax* hypnozoites with primaquine after delivery would be beneficial, but safety for the breastfeeding newborn has not been established. Clearly the management of vivax malaria in pregnancy must be improved.
